# Association between IL-6-174G/C Polymorphism and the Risk of Sepsis and Mortality: A Systematic Review and Meta-Analysis

**DOI:** 10.1371/journal.pone.0118843

**Published:** 2015-03-03

**Authors:** Jun-wei Gao, An-qiang Zhang, Wei Pan, Cai-li Yue, Ling Zeng, Wei Gu, Jianxin Jiang

**Affiliations:** State Key Laboratory of Trauma, Burns and Combined Injury, Institute of Surgery Research, Daping Hospital, Third Military Medical University, Chongqing, 400042, China; Queen Mary University of London, UNITED KINGDOM

## Abstract

**Background:**

Recent studies have reported the association between IL-6-174G/C polymorphism and sepsis. However, the results are inconclusive and conflicting. To better understand the role of IL-6-174G/C polymorphism in sepsis, we conducted a comprehensive meta-analysis.

**Methodology:**

Literature search was conducted through PubMed, Embase, Web of Knowledge databases until July 29, 2013. The pooled odds ratios (ORs) and 95% confidence intervals (CIs) were calculated using fixed- or random-effect model based on heterogeneity test in total and subgroup analyses.

**Results:**

Twenty studies on the risk of sepsis and seven studies on sepsis mortality were included. None of the results showed evidence of a significant association between IL-6-174G/C polymorphism and sepsis risk in overall analysis or subgroup analyses based on sepsis type, ethnicity, source of control and age under any genetic model (the allele comparison, the codominant, the recessive or the dominant model). Although there was a statistically significant association between IL-6-174 G/C polymorphism and sepsis-related mortality under the recessive model, the significance did not exist after Bonferroni’s correction.

**Conclusions:**

Current evidence does not support a direct effect of IL-6-174 G/C polymorphism on the risk of sepsis. In addition, there was no association between IL-6-174 G/C polymorphism and sepsis mortality after Bonferroni’s correction. Further analyses of gene-environment interactions and more studies based on larger sample size and homogeneous sepsis patients are required.

## Introduction

Sepsis is characterized by systemic inflammatory response of the host organism to the invasion of microorganisms. Despite the advances in the development of antibiotics and clinical care, sepsis remains the leading cause of death in critically ill patients. About $16.7 billion is spent in U.S. annually for sepsis with the mortality rate of 28.6% [1]. If we can find an effective method which could help us identify high-risk patients who may develop to sepsis, we could take early intervention in the high-risk patients to prevent the occurrence of sepsis [2]. Cytokines play vital roles in the regulation of host immune response, and altered expression of cytokines is proven to be associated with the development of sepsis [3]. Recently, a quantity of publications have revealed that genetic variation especially single nucleotide polymorphism of cytokines in the innate immune system may influence the risk of sepsis [4–6]. Among these cytokines, Interleukin-6 (IL-6) is one of the most important members which may be associated with sepsis risk and outcome.

IL-6 is a proinflammatory cytokine secreted by monocytes, endothelial cells and fibroblasts, and is able to stimulate B and T lymphocytes and induce fever. Some studies have indicated that IL-6 may play a key role in the inflammatory response to microbial invasion. [7]. Previous studies revealed that high IL-6 level was associated with increased severe sepsis mortality and risk. [8–11]. In addition, IL-6 has been investigated among other infection markers to improve the diagnostics of suspected neonatal infection[12]. In the other hand, IL-6 signal pathway was essential to systemic inflammation, which has been demonstrated in IL-6-deficient mice [13]. These evidences suggest that IL-6 may be an appealing candidate gene for sepsis.

The human IL-6 gene is mapped to chromosome 7p21 region and contains several single nucleotide polymorphisms. The G to C polymorphism at position-174 of the IL-6 gene (rs1800795) influences poor outcomes in a number of inflammatory diseases [14,15]. Furthermore, Fishman et al. and Kilpinen et al. found that IL-6–174G/C polymorphism caused differential activity in promoter constructs, which influenced the plasma level of IL-6 [16,17]. Although Ahrens et al. [18] found that IL-6–174G/C polymorphism increased the risk of sepsis, several studies [19–23] revealed no significant association. In 2008, Chauhan et al. [24] tried to clarify the association between IL-6–174G/C polymorphism and the risk of sepsis among very low birth weight(VLBW) infants. However, only six studies were included in the meta-analysis. Recently, increasing studies have been conducted to evaluate the association between IL-6–174G/C polymorphism and sepsis. However, the results were not consistent and remained inconclusive, which may be explained by potential limitations of studies, such as sample size and the source of control [25]. Therefore, we performed the meta-analysis based on the published papers to clarify the association between IL-6–174G/C polymorphism and sepsis.

## Methods

### Publication Search

A systematic literature search was conducted in PubMed, Embase and Web of Knowledge databases up to July 29 2013. The combination of terms “Interleukin-6” or “IL-6”; “sepsis” “septic shock” or “septicemia”; and “polymorphism” “variation” “mutation” or “genotype” was used without language restriction and publication date. Relevant studies were retrieved, and their references were checked to find other relevant publications. Authors were contacted to obtain related data not revealed in original articles.

### Inclusion and Exclusion Criteria

The inclusion criteria were: (1) evaluation of the association between IL-6–174G/C polymorphism and sepsis; (2) independent cohort or case-control studies; (3) the number or frequency of genotypes was provided in studies or obtained by contacting the authors. The exclusion criteria were: (1) studies with insufficient information, such as genotype number not reported; (2) review, comment or abstract. For studies with overlapping samples, only the one with the largest sample size was included. When an article reported the results on different ethnicities, we treated them as separate studies.

### Data Extraction

Two investigators conducted the data extraction independently in order to reduce personal error and ensure the reliability. Useful information was extracted as following: first author’s name, publication year, country, ethnicity, source of control, age, sepsis type, number or frequency of cases and controls for each IL-6–174G/C genotype. Hardy-Weinberg equilibrium was tested based on provided data. Predesigned standard data forms were used for information collection. Disagreement was resolved by discussion.

### Statistical Analysis

Because the model of inheritance of sepsis is unknown, we conducted the meta-analysis with the allele comparison model (C vs. G), the codominant model (CC vs. GG), the recessive model (CC vs. GG/GC) and the dominant model (CC/GC vs. GG). The association between IL-6–174G/C polymorphism and sepsis was evaluated by pooled odds ratio (OR) and 95% confidence interval (CI). Z test was carried out to evaluate the statistical significance of pooled ORs. In addition to overall analyses, subgroup analyses were performed according to sepsis type (the definitions according to the ACCP/SCCM guidelines [26]), ethnicity, age group, source of control and healthy adult control group under each of the four genotype models. Heterogeneity across studies was assessed by I^2^ value and Chi-square based Q-test. P>0.10 for Q-test or I^2^ value less than 50% revealed no obvious heterogeneity across studies, allowing to use a fixed effects model (the Mantel-Haenszel method); otherwise, a random effects model was selected (the DerSimonian and Laird method). Galbraith plots were performed to investigate the source of between-study heterogeneity. Sensitivity analyses were performed through removing each study in turn to evaluate the stability of results. Publication bias was examined by Begg’s funnel plot qualitatively (the more symmetrical the lower risk of publication bias) and Egger’s test quantitatively. At last, Bonferroni’s correction was conducted to control the type I error rate in the meta-analysis of mortality. All statistical analyses were performed by using Revman 5.2 software (Nordic Cochrane Center, Copenhagen, Denmark) and STATA 12.0 software (STATA Corp, College Station, TX).

## Results

### Study Characteristics

**Fig 1:**
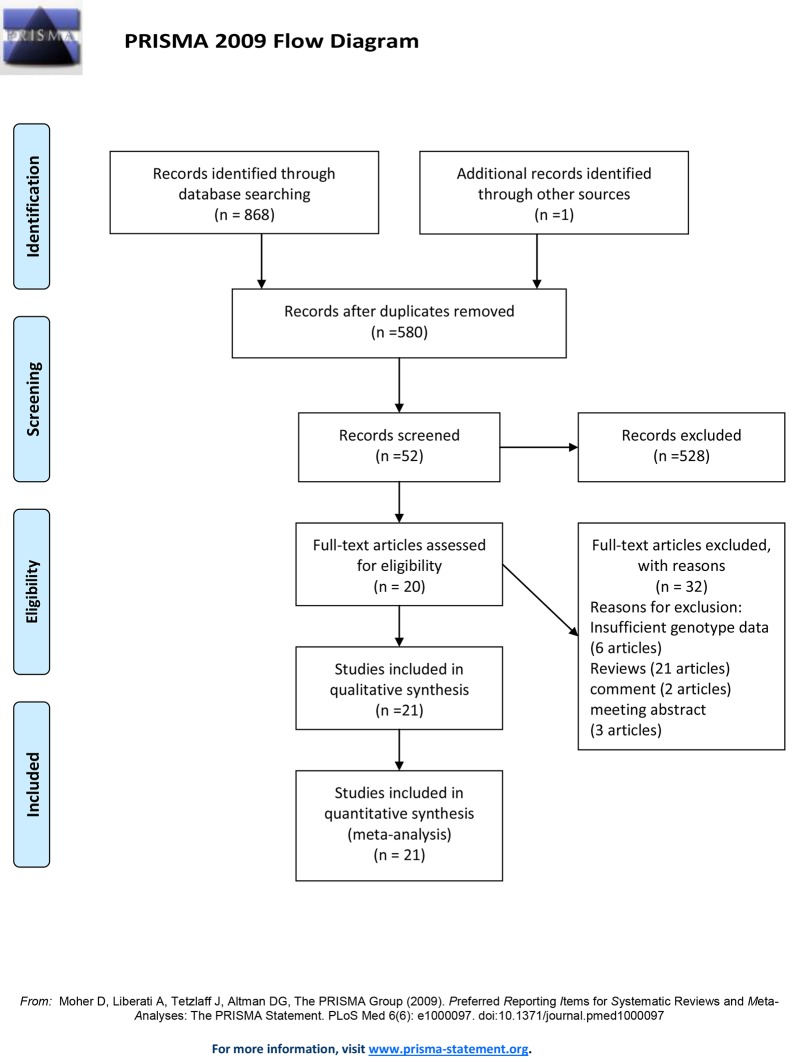
Flow diagram of study identification.

A total of 868 records were identified by searching three databases (195 from PubMed, 381 from Embase and 292 from Web of Knowledge) and 1 record was identified through references. After excluding 289 duplications, 528 records were removed for their unmatched titles or abstracts. After reading the full text of the remaining 52 records, 32 records (6 records with insufficient genotype data, 21 reviews, 2 comments and 3 meeting abstracts) were excluded. Finally, 20 records (21 studies) were included in our meta-analysis [18–23,27–40] (Flow diagram in Fig. 1). Fourteen studies were about the risk of sepsis, one study was about sepsis mortality and 6 studies were about both sides. The characteristics of the included studies are shown in Table 1. Among the 20 studies about sepsis risk, fourteen studies were consistent with Hardy-Weinberg equilibrium testing. Fourteen studies were conducted in European population, two studies were performed in African population, one study was conducted in Asian population and the others were performed in mix population. Studies were classified as sepsis (fourteen studies), severe sepsis (two studies), septicemia (two studies) and mixed (two studies). Ten records (eleven studies) were performed in adult [19–23,27,29,31,34,35], and the others were in pediatric populations [18,28,30,32,33,36–39]. All of the seven studies about sepsis mortality focused on non-Asian population. We removed the article conducted by Shimada et al. [35], because there was no mutation in the study population.

**Table 1:** Characteristics of the studies included in the meta-analysis.

				Source of	Age	Sepsis			Case			Control			
First author	Year	Country	Ethnicity	control	group	type	Case	Control	GG	GC	CC	GG	GC	CC	HWE
Schluter	2002	Germany	European	critical ill	Adult	sepsis	50	276	13	25	12	91	131	54	yes
Treszl	2003	Hungary	European	VLBW	Neonatal	sepsis	33	70	18	13	2	34	29	7	yes
Harding	2003	UK	European	preterm infants	Neonatal	septicemia	51	106	24	27^a^		30	76^a^		NA
Balding	2003	Ireland	European	healthy	Adult	sepsis	183	389	59	97	27	123	198	68	yes
Balding	2003	Ireland	European	survivors	Adult	non-survivors	25	158	13	10	2	46	87	25	yes
Barber	2004	America	Mix^1^	critical ill	Adult	severe sepsis	36	123	17	19^a^		69	54^a^		NA
Ahrens	2004	Germany	European	VLBW	Neonatal	sepsis	50	306	24	21	5	97	177	32	no
Sipahi	2006	Turkey	European	healthy	Pediatric	severe sepsis	44	77	26	14	4	52	19	6	no
Sipahi	2006	Turkey	European	survivors	Pediatric	non-survivors	15	29	9	5	1	17	9	3	no
Gopel	2006	Germany	European	VLBW	Neonatal	sepsis	97	320	29	50	18	128	143	49	yes
Baier study1	2006	America	African	VLBW	Neonatal	sepsis	114	119	94	18	2	110	9	0	yes
Baier study1	2006	America	African	survivors	Neonatal	non-survivors	11	103	10	1	0	84	17	2	yes
Baier study2	2006	America	European	VLBW	Neonatal	sepsis	31	26	12	16	3	7	16	3	yes
Baier study2	2006	America	European	survivors	Neonatal	non-survivors	3	28	1	1	1	11	15	2	yes
McDaniel	2007	America	Mix^1^	critical ill	Adult	sepsis	31	37	28^b^		3	29^b^		8	NA
Sabelnikovs	2008	Latvia	European	survivors	Adult	non-survivors	44	59	10	21	13	20	32	7	yes
Reiman	2008	Finland	European	preterm infants	Neonatal	septicemia	11	96	4^b^		7	78^b^		18	NA
Shalhub	2009	America	European	critical ill	Adult	Mix^2^	147	451	39%^c^			41%^c^			yes
Abdel-Hady	2009	Egypt	African	healthy	Neonatal	sepsis	54	71	17	26	11	28	32	11	yes
Abdel-Hady	2009	Egypt	African	survivors	Neonatal	non-survivors	13	41	2	5	6	15	21	5	yes
Sole-Violan	2010	Spain	European	CAP	Adult	Mix^2^	321	817	141	144	36	392	341	84	yes
Davis	2010	America	European	healthy	Adult	sepsis	23	52	10	11	2	21	22	9	yes
Carregaro	2010	Brazil	Mix^1^	healthy	Adult	sepsis	97	207	49	39	9	94	96	17	yes
Shimada	2011	Japan	Asian	critical ill	Adult	sepsis	123	101	123	0	0	101	0	0	yes
Shimada	2011	Japan	Asian	survivors	Adult	non-survivors	21	102	21	0	0	102	0	0	yes
Martin-Loeches	2012	Spain	European	healthy	Adult	sepsis	1227	953	581	516	130	438	413	102	yes
Palumbo	2012	Italy	European	critical ill	Adult	sepsis	16	26	14^b^		2	22^b^		4	yes

NA, not available,

CAP: Community Acquired Pneumonia,

VLBW: very low birth weight,

Mix^1^: European, African and so on,

Mix^2^: severe sepsis and septic shock,

^a^ represents the number of GC+CC genotype,

^b^ represents the number of GG+GC genotype,

^c^ represents the frequency of the C allele,

HWE: Hardy-Weinberg equilibrium.

### Quantitative Data Synthesis

Eighteen records (nineteen studies) [18–23,27–34,36–39] determined the association between IL-6–174G/C polymorphism and sepsis risk after removing the record conducted by Shimada et al. (2011). Under all of the four genetic models, there was no significant association between IL-6–174G/C polymorphism and sepsis risk for overall analysis (for C vs. G: OR = 1.01, 95%CI 0.93–1.09, P = 0.82; for CC vs. GG: OR = 1.04, 95%CI 0.87–1.25, P = 0.68; for CC vs. GC/GG: OR = 1.04, 95%CI 0.88–1.23, P = 0.64; for CC/GC vs. GG: OR = 1.02, 95%CI 0.85–1.23, P = 0.81;)(Table 2). After removing studies that were not consistent with Hardy-Weinberg equilibrium testing, the results remained unchanged (data not shown). We conducted further meta-analyses stratified by sepsis type, ethnicity, source of control, age and healthy adult control under four genetic models. Similarly, the results of the subgroup analyses showed that this polymorphism was not significantly associated with sepsis risk. As there were less than three studies,subgroup analyses about other sepsis types, ethnicities and source of control were not performed (Table 2).

**Table 2:** Summary of meta-analysis results.

		Test of association				Heterogeneity		
Groups	Studies	OR[95%CI]	p value	Model	Z	X^2^	p value	*I* ^2^(%)
Total studies								
C vs. G	14	1.01[0.93–1.09]	0.82	FE	0.23	18.57	0.14	30
CC vs. GG	13	1.04[0.87–1.25]	0.68	FE	0.42	8.69	0.73	0
CC vs. GC/GG	16	1.04[0.88–1.23]	0.64	FE	0.47	15.58	0.41	4
CC/GC vs. GG	15	1.02[0.85–1.23]	0.81	RE	0.24	25.90	0.03	46
Subgroup								
Sepsis								
C vs. G	11	0.99[0.91–1.09]	0.90	FE	0.12	15.85	0.10	37
CC vs. GG	11	1.00[0.82–1.23]	0.97	FE	0.04	8.09	0.62	0
CC vs. GC/GG	13	0.99[0.82–1.19]	0.89	FE	0.14	6.67	0.88	0
CC/GC vs. GG	11	1.01[0.82–1.26]	0.90	RE	0.12	17.31	0.07	42
Neonatal infant								
C vs. G	6	1.09[0.77–1.55]	0.63	RE	0.48	12.58	0.03	60
CC vs. GG	6	1.20[0.77–1.86]	0.42	FE	0.80	5.08	0.41	2
CC vs. GC/GG	7	1.37[0.94–2.01]	0.10	FE	1.62	9.12	0.17	34
CC/GC vs. GG	7	0.94[0.57–1.55]	0.80	RE	0.25	20.49	0.002	71
Adult								
C vs. G	7	0.99[0.90–1.08]	0.78	FE	0.28	4.31	0.64	0
CC vs. GG	6	1.00[0.82–1.23]	0.97	FE	0.04	2.95	0.71	0
CC vs. GC/GG	8	0.97[0.81–1.18]	0.78	FE	0.28	4.12	0.78	0
CC/GC vs. GG	7	1.01[0.89–1.14]	0.87	FE	0.17	4.51	0.61	0
European								
C vs. G	11	1.02[0.65–1.58]	0.94	RE	0.07	111.40	<0.00001	93
CC vs. GG	10	1.01[0.84–1.23]	0.89	FE	0.14	6.66	0.67	0
CC vs. GC/GG	12	1.04[0.87–1.24]	0.70	FE	0.39	12.21	0.35	10
CC/GC vs. GG	11	0.97[0.79–1.18]	0.73	RE	0.34	18.42	0.05	46
Healthy control								
C vs. G	6	0.97[0.88–1.08]	0.59	FE	0.54	2.82	0.73	0
CC vs. GG	6	0.96[0.76–1.21]	0.72	FE	0.36	0.72	0.81	0
CC vs. GC/GG	6	0.97[0.78–1.20]	0.76	FE	0.31	2.17	0.83	0
CC/GC vs. GG	6	0.96[0.84–1.11]	0.60	FE	0.52	2.62	0.76	0
VLBW control								
C vs. G	5	1.05[0.68–1.62]	0.82	RE	0.22	12.09	0.02	67
CC vs. GG	5	1.11[0.68–1.82]	0.67	FE	0.43	4.66	0.32	14
CC vs. GC/GG	5	1.12[0.71–1.77]	0.62	FE	0.50	2.01	0.73	0
CC/GC vs. GG	5	1.01[0.54–1.88]	0.97	RE	0.003	14.31	0.006	72
Healthy adult								
C vs. G	4	0.95[0.85–1.06]	0.36	FE	0.92	0.44	0.93	0
CC vs. GG	4	0.92[0.72–1.17]	0.51	FE	0.67	0.89	0.83	0
CC vs. GC/GG	4	0.94[0.75–1.18]	0.59	FE	0.54	1.43	0.70	0
CC/GC vs. GG	4	0.94[0.81–1.08]	0.37	FE	0.90	0.38	0.94	0
Mortality analysis								
C vs. G	6	1.19[0.61–2.33]	0.61	RE	0.51	14.38	0.01	65
CC vs. GG	6	1.86[0.55–6.28]	0.32	RE	1.00	10.92	0.05	54
CC vs. GC/GG	6	1.92[1.06–3.84]	0.03	FE	2.16	8.45	0.13	41
CC/GC vs. GG	6	0.95[0.59–1.52]	0.82	FE	0.23	8.86	0.11	44

VLBW: very low birth weight.

**Fig 2:**
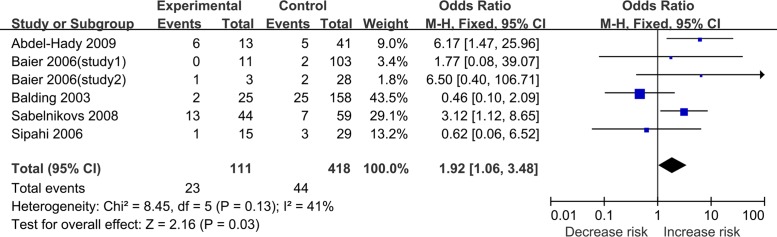
Forest plot of sepsis mortality associated with IL-6–174G/C polymorphism under the recessive model.

There were five records (six studies) [36–40] about sepsis-related mortality. Of the total 652 patients, 132 patients died finally. There was a statistical association between IL-6–174 G/C polymorphism and sepsis-related mortality under the recessive model (for CC vs. GC/GG: OR = 1.92, 95%CI 1.06–3.84, P = 0.03) (Fig. 2, Table 2). However, this polymorphism was no longer significantly associated with sepsis-related mortality after Bonferroni’s correction (data not shown). Furthermore, we found no statistical association under the other three models.

### Heterogeneity Analysis

**Fig 3:**
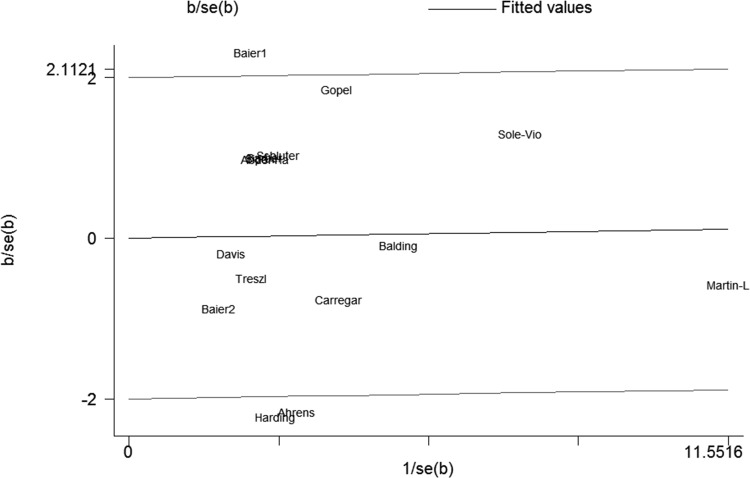
Galbraith plot of IL-6–174G/C polymorphism and the risk of sepsis under the dominant model.

There was no obvious heterogeneity for overall analysis about sepsis risk except the dominant model (P = 0.03). Although subgroup analyses were conducted, the heterogeneity did not decrease effectively except for adult subgroup analysis. We explained the heterogeneity by Galbraith plot. Three studies performed by Ahrens et al. [18], Baier et al. [38] (study 1) and Harding et al. [33] respectively were outliers in the Galbraith plot under the dominant model (Fig. 3). After removing them, the between-study heterogeneity decreased effectively. However, there was no significant association between IL-6–174G/C polymorphism and sepsis risk.

**Fig 4:**
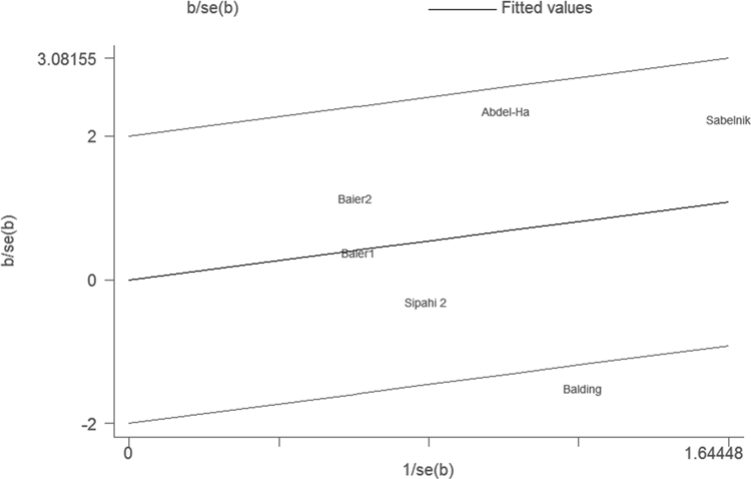
Galbraith plot of IL-6–174G/C polymorphism and sepsis mortality under the codominant model.

**Fig 5:**
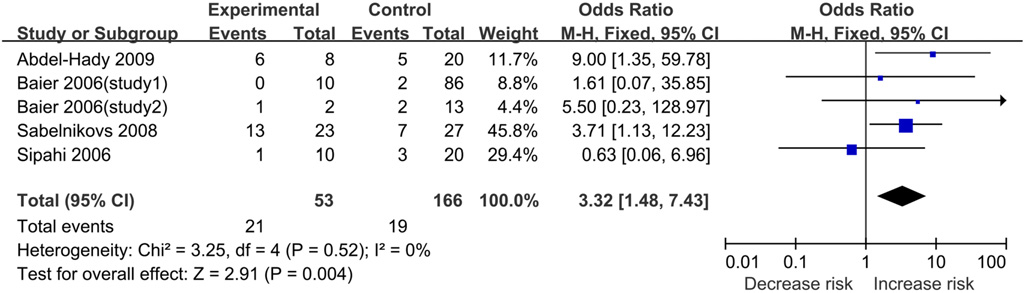
Forest plot of sepsis mortality associated with IL-6–174G/C under the allelic genetic model after deleting the study conducted by Balding et al.

For sepsis mortality, obvious heterogeneity also existed under the allele comparison model and the codominant model (P = 0.01 and P = 0.05) (Table 2). Galbraith plots were used to explore the source of heterogeneity. Studies performed by Abdel-Hady et al. [36] and Balding et al. [39] were out of the bounds under the allele comparison model. After excluding these studies, the between-study heterogeneity effectively decreased, but the result changed little. Under the codominant model, the study conducted by Balding et al. [39] was out of the bounds (Fig. 4). After removing the study, the heterogeneity decreased notably and there was a significant association between IL-6–174G/C polymorphism and sepsis-related mortality under the codominant model (for CC vs. GG: OR = 3.32, 95%CI 1.48–7.43, P = 0.004) (Fig. 5). However, this polymorphism was no longer significantly associated with sepsis-related mortality after Bonferroni’s correction (data not shown).

### Sensitivity Analysis

Sensitivity analyses were carried out in order to examine the results of our meta-analysis through removing each study sequentially. No obvious changes were found in the results, which confirmed our results were stable under the four models.

### Publication Bias

**Fig 6:**
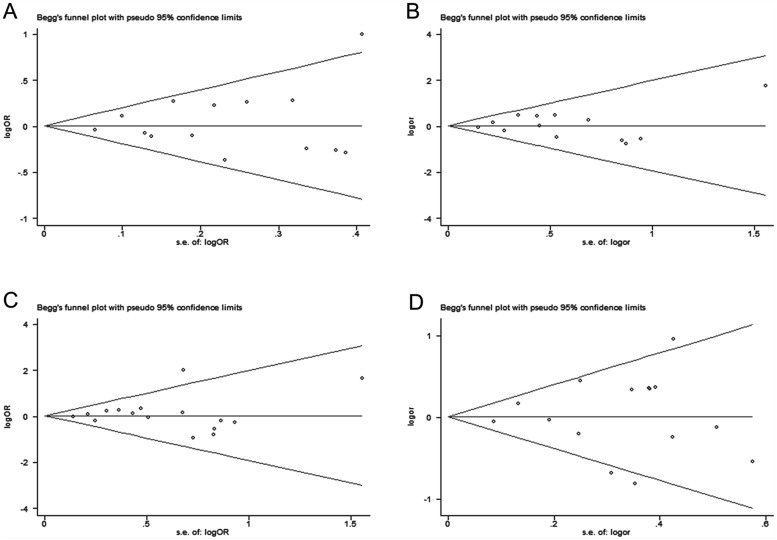
Funnel plots of IL-6–174G/C polymorphism and the risk of sepsis to assess publication bias under different models (A: the allele comparison model; B: the codominant model; C: the recessive model; D: the dominant model).

Egger’s test and Begg’s funnel plot were used to evaluate publication bias quantitatively and qualitatively respectively. Slight asymmetry was found in all of the four plots (Fig. 6). Egger’s test didn’t exhibit obvious publication bias under all models quantitatively (for the allele comparison model P = 0.581; for the codominant model P = 0.824; for the recessive model P = 0.631; for the dominant model P = 0.852). The examination of publication bias about sepsis-related mortality was not conducted because only several studies were included.

## Discussion

To date, about twenty studies have been reported to evaluate the association between IL-6–174G/C polymorphism and sepsis [18–23,27–40]. However, the results were inconsistent and most studies [19–23,29,31,32,35–37,39] failed to identify the association. A meta-analysis performed by Chauhan et al. suggested a negative association between IL-6–174G/C polymorphism and sepsis risk among pediatric population in 2008. Here, we have extended the meta-analysis with a larger sample size and different subgroups. We believe our result is more convincing because fourteen studies were added and four genetic models were used in our meta-analysis. In addition, we extended our meta-analysis to evaluate mortality.

In our meta-analysis, we evaluated the association between IL-6–174G/C polymorphism and sepsis under the allele comparison, codominant, recessive and dominant model, respectively. There was no association between IL-6–174G/C polymorphism and sepsis risk. Subgroup analyses according to ethnicity, sepsis type, source of control and healthy adult control still showed no obvious association between this polymorphism and sepsis risk. There were only one study on Asians and no mutation was found in its study population. More studies in Asian population are needed to estimate the effect of IL-6–174G/C polymorphism on sepsis risk. Although a statistical association was found between IL-6–174G/C polymorphism and sepsis mortality under the recessive model (for CC vs. GC/GG: OR = 1.92, 95%CI 1.06–3.84, P = 0.03), this was not maintained after Bonferroni’s correction. Meanwhile, we did not find statistical association between this polymorphism and sepsis mortality under other three models. Therefore, there was only a moderate association between IL-6–174G/C polymorphism and sepsis mortality under the recessive model. Taken together, more studies are needed to evaluate the effect of this polymorphism on sepsis mortality.

The between-study heterogeneity in our meta-analysis still existed in both overall comparisons and subgroup analyses. The results revealed that studies conducted by Baier et al. [38] (study1), Ahrens et al. [18] and Harding et al. [33] may be the major source of heterogeneity in overall comparisons. There was no significant association between IL-6–174G/C polymorphism and sepsis risk after removing these studies even though the heterogeneity decreased effectively. In contrast, there was a statistical association between IL-6–174G/C polymorphism and sepsis-related mortality under the codominant model with no obvious heterogeneity after excluding the study performed by Balding et al. [39]. However, this polymorphism was no longer significantly associated with sepsis-related mortality after Bonferroni’s correction. Recently, some researchers have found the association between the level of plasma IL-6 and early mortality (before day 7). However, we could not collect the associated information from previous studies for the limited data. Therefore, more studies especially about sepsis early mortality should be included to evaluate the effect of this polymorphism on sepsis-related mortality. Furthermore, we performed sensitivity analysis. The stability of our results was confirmed by removing each study with the unchanged results.

The results may be influenced by the publication bias which could make false-negative results suppressed or false-positive results magnified. Although Begg’s funnel plots were slightly asymmetrical, Egger’s test revealed no obvious publication bias for sepsis risk and mortality in our meta-analysis. Therefore, more studies were needed to verify the results of our meta-analysis. All of the results should be interpreted cautiously.

Even though there were more than twenty studies in our meta-analysis, limitations in our meta-analysis should be pointed out. Firstly, the number of studies was moderate especially in subgroup analyses, thus limiting the interpretation of results in our study. Secondly, the study population in our meta-analysis focused on Europeans and Americans. Our conclusion may be not suitable for Asian. Thirdly, sepsis is a complex clinical syndrome related to systemic inflammation supposed to be generated by infection. Such a syndrome concerns very different pathogens with different virulence, different location and different sensitivity to antibiotics. All aspects above add population heterogeneity. The mortality and potentially the risk of sepsis are largely influenced by medical environment particularly the so called “co-morbidity”, such as chronic disease and resistant bacteria. However, we could not make all factors matched for the limited information, which may influence the results. Fourthly, we could not ensure the consistency in control samples of all studies, which may play a crucial role in drawing a conclusion exactly. Finally, we could not address the interactions of genes for the lack of the related information.

In conclusion, the IL-6–174G/C polymorphism may not be associated with the risk of sepsis. In addition, there was no association between IL-6–174 G/C polymorphism and sepsis mortality after Bonferroni’s correction. Further analyses of gene-environment interactions and more studies based on larger sample size and homogeneous sepsis patients are required.

## Supporting Information

S1 PRISMA Checklist The PRISMA Checklist for our meta-analysis. (DOC)
